# Editorial: Psychological factors in physical education and sport – volume IV

**DOI:** 10.3389/fpsyg.2025.1558668

**Published:** 2025-02-07

**Authors:** Carla Chicau Borrego, Manuel Gómez-López, Marianna Alesi, David Manzano-Sánchez

**Affiliations:** ^1^Sport Sciences School of Rio Maior, Polytechnic Institute of Santarém - Sport Physical Activity and Health Research & INnovation Center, Santarem, Portugal; ^2^Department of Physical Activity and Sport, Faculty of Sports Sciences, University of Murcia, Murcia, Spain; ^3^Department of Psychology, Educational Sciences, and Human Movement, University of Palermo, Palermo, Italy; ^4^Department of Education, Faculty of Education Sciences, University of Almería, Almería, Spain

**Keywords:** motivation, education, sport, physical education, psychological wellbeing

This Research Topic is the fourth volume of Psychological Factors in Physical Education and Sport and aims to collect the latest research on factors influencing physical and psychological wellbeing and adherence to physical activity in the context of sports and physical education classes. The research and development of these psychological variables can lead to:

## Fostering a supportive and inclusive environment that promotes lifelong fitness and health

Belando-Pedreño et al. designed and implemented educational programs at different stages of human development that promote emotional competencies, interpersonal competencies, adequate levels of healthy physical activity, adherence to the Mediterranean diet, and a more active physical and social lifestyle on a physical and social level. In order to identify effective strategies to improve the quality of physical education systems, Jaeuk et al. undertook research that examines the nuances of educational culture, specifically focusing on the psychological experiences of early adolescents within physical education classes. Within the same context, research by Gomez-Paniagua et al. aimed to evaluate the levels of Life Satisfaction of Physical Education students in primary and secondary education in a region in southwestern Spain (Extremadura), analyzing the possible influences of gender and environment of residence.

Positive attitudes toward physical activity have consistently been linked to healthy behaviors. The study by Nie aimed to contribute to the understanding of the relationship between attitudes, self-generated information, and physical activity among university students. It also sought to enhance knowledge of the effectiveness of attitude change in the domain of exercise and identify the mechanisms through which this change occurs, with the goal of promoting exercise behavior in the context of sports.

Globally, there is a persistent public health concern regarding the high prevalence of overweight and obesity. Hu et al. investigated the developmental of obese junior high school students, focusing on the characteristics of physical education is crucial. Engaging in regular physical activity, according to Wu et al., positively predicts school adaptation among Chinese junior high school students through resilience and coping styles. Defined as a problematic behavior, mobile phone dependence was analyzed by Mei et al. in a review article, which explored the individual and environmental factors, neurobiological mechanisms, and theoretical models associated with this Research Topic in athletes.

The fundamental role of motivation: Recognizing the type of motivation that drives athletes and participants is essential for coaches and educators in crafting training programs that tap into an individual's inner drive, while also fostering a sense of external reward and recognition.

Grounded in self-determination theory, Kruse et al. a examined a multidimensional measurement of support of the basic psychological needs and the individual and combined effects on the development of intrinsic motivation and perceived competence in physical education. In another study, Kruse et al. b presented a measurement model of instructional quality that has proven to be one of the strongest predictors of educational outcomes, such as achievement and motivation. Findings by Yu et al. contributed to the theoretical frameworks of sports psychology and educational psychology by emphasizing the pivotal role of leisure satisfaction and mental toughness in the psychological development of individuals.

Psychological theories and constructs may not be universally applicable due to cultural differences. What is considered a psychological need in one culture may not hold the same significance in another. Researchers must be cautious when generalizing findings from one country to another. Cultural factors can influence an athlete's perception of their needs and how they experience fulfillment (Melesse et al.).

Managing Stress and Anxiety. Stress and anxiety are natural reactions to the competitive nature of sports, but how athletes and coaches manage these emotions can drastically affect their performance. Zhou et al. examined how the characteristics of competitive anxiety impact sports performance across different phases of competitive preparation, alongside its correlation with trait anxiety. Exploring sports participation, muscle-strengthening exercise, and active commuting with comorbidity of depression and anxiety among Chinese children and adolescents, Feng et al. concluded that sports can help relieve anxiety and depression.

Mind wandering (MW) among athletes during training and competition can lead to poor performance. Focus and concentration are often the deciding factors in close competitions. However, Li et al. showed that MW has also been found to have positive effects. With a sample of college students, Liu et al. examined the association between physical activity and negative emotions, specifically depression, anxiety, and stress. In addition, they explored the mediating effects of psychological resilience and coping styles to offer theoretical and practical insights for mitigating students' negative emotions.

Coaches play an important role in sports. Yu and Cheng concluded that reducing emotional exhaustion and negative coping, enhancing emotional support, and improving the academic titles of coaches can help reduce job pressure and occupational burnout among competitive sports coaches. Ferreira et al., in a systematic review, emphasized the importance of social support for coaches in both their personal and professional lives, noting its positive effects and the negative consequences of its absence.

Leadership in sports involves developing individuals, fostering teamwork, and creating a positive environment that promotes growth and success. Centered on coaches, Zhu et al. conducted a systematic review and meta-analysis of Chinese coach leadership and its impact on athlete satisfaction and cohesion. The autonomy-supportive coaching style is recognized for its positive impact on athletes' wellbeing and performance. Su et al. aimed to integrate the autonomy-supportive and laissez-faire coaching styles within the same measurement framework .

On the field, when faced with complex foul situations, referees must make swift and accurate decisions, Wang et al., in the framework of decision-making, examined how soccer referees make decisions about issuing yellow cards for fouls.

Based on these theoretical premises, this Research Topic aimed to address essential questions and collect the most recent research on factors influencing physical and psychological wellbeing, as well as adherence to PA, in the context of physical education classes and sports.

